# Hybrid TiO_2_-ZnO Nanomaterials Prepared Using Laser Ablation in Liquid

**DOI:** 10.3390/ma13030719

**Published:** 2020-02-05

**Authors:** Neli Mintcheva, Shigeru Yamaguchi, Sergei A. Kulinich

**Affiliations:** 1 Research Institute of Science and Technology, Tokai University, Hiratsuka, Kanagawa 259-1292, Japan; 2 Department of Chemistry, University of Mining and Geology, 1700 Sofia, Bulgaria; 3 Department of Physics, Tokai University, Hiratsuka, Kanagawa 259-1292, Japan; shigeru@keyaki.cc.u-tokai.ac.jp; 4 Department of Mechanical Engineering, Tokai University, Hiratsuka, Kanagawa 259-1292, Japan; 5 School of Natural Sciences, Far Eastern Federal University, 690041 Vladivostok, Russia

**Keywords:** TiO_2_ nanospheres, ZnO nanorods, hybrids TiO_2_-ZnO, Ti^3+^ doped titania, XPS

## Abstract

Hybrids of semiconductor nanomaterials often demonstrate properties that are superior to those of their components. In this study, we prepared hybrid nanomaterials of TiO_2_ and ZnO, which are among the most actively studied semiconductors, by means of millisecond-pulsed laser and analyzed how their morphology, particle size, and surface composition depend on preparation conditions. A series of nanomaterials were obtained via sequentially ablating Zn and Ti metal plates (in different sequences) in water, while laser pulses of lower (2.0 J/pulse) and higher (5.0 J/pulse) energy were applied. The properties of laser-produced hybrid TiO_2_-ZnO nanomaterials were shown to be governed by experimental conditions such as laser pulse width, pulse peak power, and reaction media (either pure water or colloid with nanoparticles). The morphology revealed nanospheres of TiO_2_ that decorate nanorods of ZnO or flower-like aggregates of zinc oxide. Intriguingly, after extended ablation time, titania was found to be self-doped with Ti^3+^ and Ti^2+^ ions, and the contribution of lower oxidation states of titanium could be controlled by the applied laser pulse energy. The physicochemical characteristics of hybrid nanomaterials were compared with pure ZnO and TiO_2_ prepared under the same laser conditions.

## 1. Introduction

Laser ablation in liquid (LAL) is a convenient, reliable, and efficient technique to produce various types of colloidal nanomaterials, from monometallic nanoparticles (NPs) to functionalized composites [1,2,3,4,5,6,7,8,9]. This method is considered to be an environmentally friendly approach, as it uses a minimum volume of solvents (as media) and reagents to produce NPs with a clean surface, without generating big amounts of chemical waste or side products [1,2,3,4,5,6,7,8,9,10,11,12]. It is a versatile method that permits preparing a large number of diverse metal oxide, sulfide, and carbide NPs via laser-induced chemical reactions that take place in a limited zone of the reaction vessel [3,4]. In a typical procedure, the laser beam is focused on a metal target immersed in liquid, producing plasma, vapor, or molten metal drops that then react with the liquid medium to form particular compounds and grow as NPs [5]. In case of chemically inert metals, no reaction occurs, and bare metal NPs are produced [6,7,8,9]. The extremely high temperature gradient and quenching rates created in the reaction zone during LAL often lead to the formation of metastable phases or unique morphologies of nanomaterials, as well as defect-rich surfaces, which are the main reasons for the potential attractiveness of LAL-produced nanomaterials for catalysis and photocatalysis [7,8,9,10], optics and optoelectronics, sensing, and biomedical applications [1,2,3,4,5,6,11,12,13,14,15].

LAL is well-known as a convenient laboratory-scale method for the fabrication of metal oxide NPs, including TiO_2_ and ZnO, which are widely used and investigated semiconductor nanomaterials with respect to their photocatalytic, photovoltaic, antibacterial, and sensing properties [3,4,16,17,18,19,20,21,22,23,24,25,26,27,28,29,30,31,32,33,34,35,36,37,38,39,40,41,42,43,44,45]. Although laser-produced TiO_2_ nanomaterials were found to be often amorphous and hydroxyl-rich, they demonstrated good photocatalytic activity owing to the high density of oxygen vacancies and Ti^3+^ ions on the surface, small NP size, and presence of nonstoichiometric titanium oxides [16,17,18,19,20,21]. If necessary, the prepared TiO_x_ colloids could be further modified by laser post-irradiation that could change their size, morphology, structure, and certain properties [16,17,18,19,20,21,22]. Nanomaterials of TiO_2_ with different phase composition were reported by different groups, depending on the applied laser, energy fluence, and liquid media. For example, ablation of Ti metal by IR-nanosecond-pulsed laser in water and methanol was reported to produce anatase [23,24]. When the second harmonic (532 nm) of Nd:YAG nanosecond laser was applied in water, it gave a mixture of anatase and rutile [25], while the presence of surfactants was found to favor the growth of the rutile phase [16,26]. Boutinguiza et al. achieved control over the predominant phase composition of TiO_2_ NPs. By using different solvents, their LAL experiments with continuous-wave laser in ethanol and water provided mainly brookite and rutile as dominant phases, respectively [27,28].

In contrast to TiO_2_, LAL-produced ZnO NPs typically have a wurtzite crystal structure, but their morphology, size, and surface defects differ depending on liquid and laser used. In order to understand and control such nanostructures, Reich et al. explored the mechanism of zinc oxidation and the dynamics of the Zn-ablation process in water by X-ray spectroscopy and imaging methods [29]. The majority of studies that used ns-pulsed lasers for ablating Zn plates in water presented the preparation of spherical ZnO NPs with different sizes and photoluminescence (PL) properties [30,31,32,33,34,35,36,37,38,39]. Although the band gap and exciton emission bands in PL spectra of most ZnO NPs prepared by means of laser with a wavelength of 1064 nm were similar, ZnO products ablated with shorter wavelengths (532 and 355 nm) demonstrated more significant changes in their particle sizes and morphologies [34,35].

To reduce the size of ZnO NPs and stabilize them in water medium, various surfactants have been used [36,37,38]. In some cases, a similar effect could be achieved by pH control of the media. For instance, He et al. obtained small, stable, and monodispersed ZnO NPs formed in acidic and basic media, which was explained by the high absolute surface charge of ZnO NPs, while at neutral pH similar NPs easily coalesced to bigger aggregates [39]. Desarkar et al. reported solid and hollow Zn/ZnO NPs that were produced by ns-laser in water [40,41]. Interestingly, ZnO nanorods with different aspect ratios depending on pulse width and pulse energy of millisecond-long pulses were obtained by ablating a Zn metal plate in water [42]. Predictably, spherical and rod-shaped ZnO NPs prepared by ns- and ms-pulsed lasers demonstrated different properties when tested as gas sensors and photocatalysts with different morphology and surface defects [43,44,45].

Enhanced sensing and photocatalytic performance was observed in case of hybrids of both laser-prepared ZnO and TiO_2_ NPs with other semiconductor oxides [46,47,48,49]. Unexpectedly, however, little work was reported so far on ZnO-TiO_2_ hybrid nanomaterials prepared via LAL, even though the method permits the preparation of hybrid nanomaterials in a relatively easy way [50]. Such hybrid nanomaterials could be attractive, e.g., for sensing, photocatalytic, catalytic, optoelectronic, and photovoltaic applications, which was one of the motivations for the present study.

Therefore, in this work, not only did we prepare new TiO_2_ and ZnO NPs using a ms-pulsed laser, but we also explored their combinations obtained with different sequences by the same laser. To our best knowledge, LAL-produced TiO_2_-ZnO hybrids have not been reported to date, which is why the structure and morphology of such novel materials were the main focus of this work. Apart from laser parameters that influence the nature and properties of the product, we also investigated the role of the reaction media on NP formation, as well as the effect of pulse energy on water-dispersed NPs during their post-irradiation stage. Two types of hybrids were produced, for which either a Ti or Zn metal plate was ablated first, followed by ablation of the other metal in the presence of already-formed TiO_2_ or ZnO NPs. For comparison, pure TiO_2_ and pure ZnO NPs were also prepared under the same laser conditions, so that we designed two series of nanomaterials denoted as the lower-energy series (TiO_2_-1, ZnO-1, ZnO/TiO_2_-1, and TiO_2_/ZnO-1) and the higher-energy series (TiO_2_-2, ZnO-2, ZnO/TiO_2_-2, and TiO_2_/ZnO-2). All the above listed materials were characterized and compared.

## 2. Materials and Methods

A Zinc plate (99.5% purity, 2-mm thick, from Nilaco, Japan) and Ti plate (99.5% purity, 0.5-mm thick, from Nilaco, Japan) were degreased in organic solvents via sonication and then ablated in deionized water. A millisecond-pulsed Nd:YAG laser (ML-2150A from Miyachi Co. Ltd., Isehara, Japan) with a wavelength of 1064 nm, pulse peak power of 1.0 kW (or 5.0 kW), pulse width of 2.0 ms (or 1.0 ms), and repetition rate of 5 Hz was applied to ablate the metal target through the side wall of a quartz cuvette. The beam was focused on the target surface by a lens with the focal length of 9.0 cm, with a spot diameter of ~150 µm. The experimental conditions and sample descriptions are given in Table 1, while more details on the setup used can be found elsewhere [42,43,44].

For simplicity, in Table 1, the samples are divided into two groups: (i) the lower-energy series, which includes samples of TiO_2_-1, ZnO-1, ZnO/TiO_2_-1, and TiO_2_/ZnO-1 and was prepared with a pulse energy of 2.0 J/pulse; and (ii) the higher-energy series, which includes samples of TiO_2_-2, ZnO-2, ZnO/TiO_2_-2, and TiO_2_/ZnO-2, all being prepared with a pulse energy of 5.0 J/pulse. The samples of TiO_2_ and ZnO were prepared from their corresponding metal plates, which were ablated in 15 mL of deionized water for 30 min. The hybrid samples of ZnO/TiO_2_ were prepared by first ablating a Zn plate in 15 mL of deionized water for 30 min, after which the Zn plate was removed and replaced with a Ti plate. The latter was thus ablated for another 30 min in a ZnO colloid. Similarly, the hybrid samples of TiO_2_/ZnO were prepared by successively ablating a Ti plate in water and then a Zn plate in the TiO_2_ colloid.

The as-prepared nanomaterials were deposited on Cu grids for transmission electron microscopy (TEM). The micrographs were taken on a Hitachi HF-2200 TEM instrument (Tokyo, Japan). The colloids were centrifuged at 16,500 rpm for 15 min, after which the separated materials were drop-cast onto Si wafers as substrates. All samples were annealed at 400 °C for 2 h and then analyzed by X-ray photoelectron spectroscopy (XPS, Quantum 2000, ULVAC-PHI, Inc., Chigasaki, Japan) and X-ray diffraction (XRD, D8 Discover, Bruker, Yokohama, Japan). UV-vis absorption spectra of freshly prepared colloids were recorded by a UV-vis spectrophotometer (model UV-2450, Shimadzu, Kyoto, Japan) in the wavelength range from 200 to 600 nm.

## 3. Results and Discussion

### 3.1. XRD Analysis

**Figure 1:**
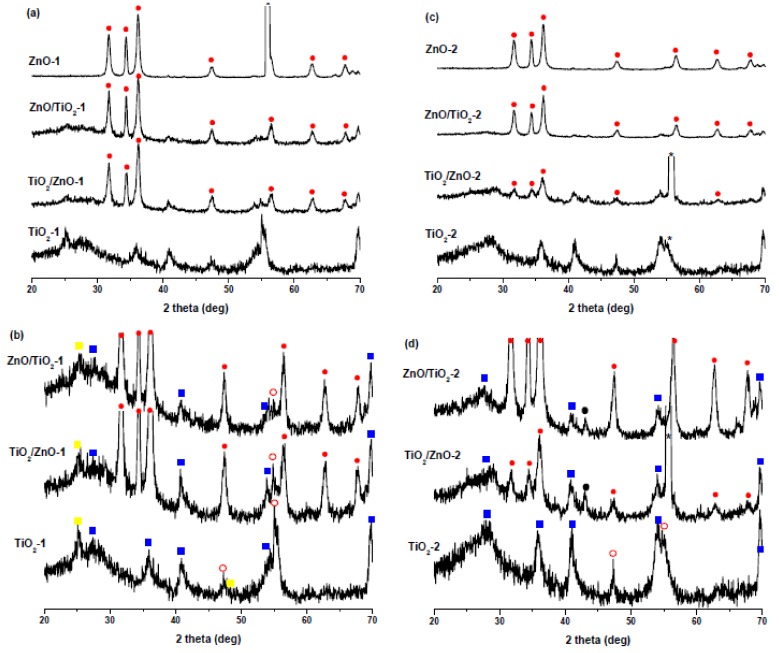
XRD patterns of (**a**) ZnO-1, ZnO/TiO_2_-1, TiO_2_/ZnO-1, and TiO_2_-1 prepared at lower pulse energy; (**b**) scaled-up patterns from panel (a) showing TiO_2_ peaks for ZnO/TiO_2_-1, TiO_2_/ZnO-1, and TiO_2_-1 in more detail. (**c**) ZnO-2, ZnO/TiO_2_-2, TiO_2_/ZnO-2, and TiO_2_-2 prepared at higher pulse energy; (**d**) scaled-up patterns from panel (c) showing TiO_2_ peaks for ZnO-2, ZnO/TiO_2_-2, TiO_2_/ZnO-2, and TiO_2_-2 in more detail. Peaks marked by asterisks are from Si substrate. Solid red circles indicate ZnO, empty red circles indicate brookite, solid yellow squares indicate anatase, solid blue squares indicate rutile, and solid black circles indicate metallic zinc.

Figure 1 shows the XRD patterns of all samples prepared at lower (Figure 1a,b) and higher pulse energy (Figure 1c,d). For clarity, the XRD patterns presented in Figure 1a,c were scaled up to better show their peaks of TiO_2_ which are much weaker than those of ZnO (Figure 1b,d). In Figure 1a,c the peaks of hexagonal wurtzite ZnO are marked with solid red circles, their positions being in good agreement with the ZnO reference (PDF 01-089-1397), as well as with XRD patterns of other LAL-produced ZnO NPs [30,31,32,36,42]. Thus, the XRD data confirm the formation of a ZnO wurtzite phase as a product of Zn-metal ablation in water and in titania suspension at both lower and higher pulse energy.

The ZnO peaks observed in the TiO_2_/ZnO-2 sample are relatively weaker, which can be explained by a somewhat hindered ablation of metallic zinc and its further oxidation to ZnO in the presence of TiO_2_ NPs. This could be because, on one hand, TiO_2_ NPs also absorb some part of laser pulses, thus reducing the efficiency of ablation. On the other hand, if molten Zn nanodroples produced by a laser beam with higher pulse energy are not small enough, they will react more slowly with H_2_O, which should produce less ZnO over the same period of time. This assumption is supported by the weak peaks of the metallic Zn phase (marked with black circles in Figure 1d) in the XRD patterns of TiO_2_/ZnO-2 and ZnO/TiO_2_-2 samples. The presence of metallic Zn as admixture in ZnO NPs prepared at higher pulse energy was also observed by Honda et al., who ablated Zn in water and ethanol by means of ms-laser [42]. 

Figure 1b,d show enlarged XRD patterns for the same TiO_2_, ZnO/TiO_2_, and TiO_2_/ZnO samples, where titania’s peaks can be seen in more detail. The ablated TiO_2_ NPs are seen to be a mixture of rutile (PDF 01-072-7374) (solid blue squares), anatase (PDF 00-001-0562) (solid yellow squares), and brookite (PDF 01-076-1934) (empty red circles) phases, with rutile being the most abundant one. The XRD pattern of TiO_2_-2 obtained at higher pulse energy consists of peaks of rutile and brookite, which is not unexpected considering that rutile is well-known to be a high-temperature phase [16,26]. These peaks are weaker and broader in the hybrid materials (samples TiO_2_/ZnO-2 and ZnO/TiO_2_-2), which probably indicates the formation of more amorphous TiO_2_ phase under prolonged laser treatment.

### 3.2. TEM and Size Distribution

**Figure 2:**
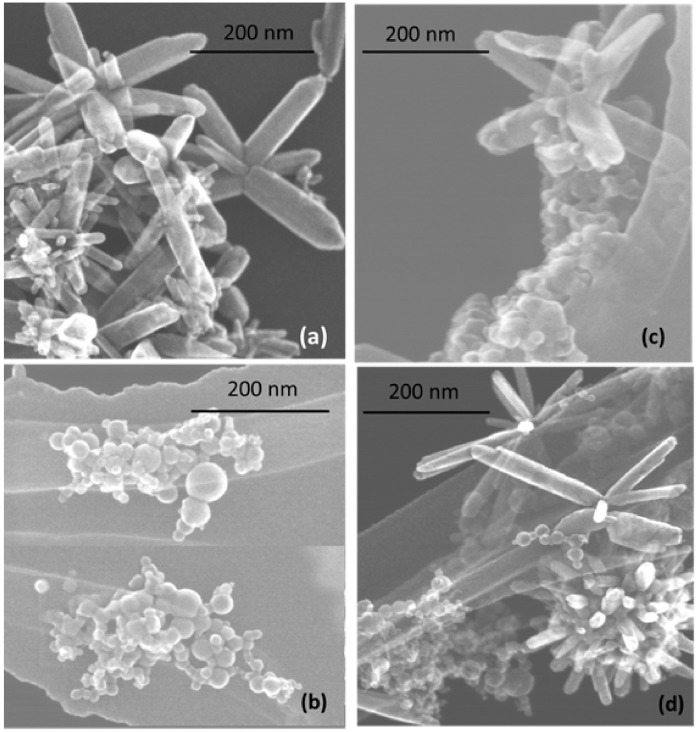
TEM images of samples prepared at lower pulse energy: (**a**) ZnO-1, (**b**) TiO_2_-1, (**c**) ZnO/TiO_2_-1, and (**d**) TiO_2_/ZnO-1. More images of the same samples can be found in Supplementary Materials, Figure S1.

**Figure 3:**
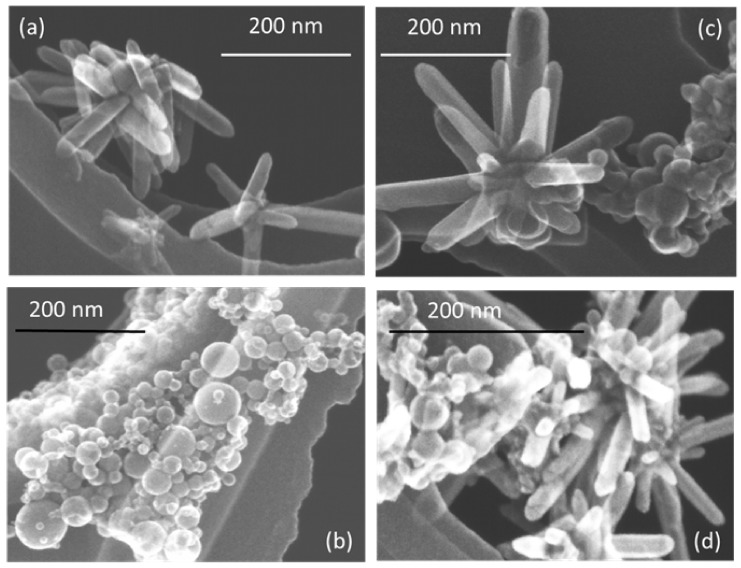
TEM images of samples prepared at higher pulse energy: (**a**) ZnO-2, (**b**) TiO_2_-2, (**c**) ZnO/TiO_2_-2, and (**d**) TiO_2_/ZnO-2. More images of the same samples can be found in Supplementary Materials, Figure S2.

**Figure 4:**
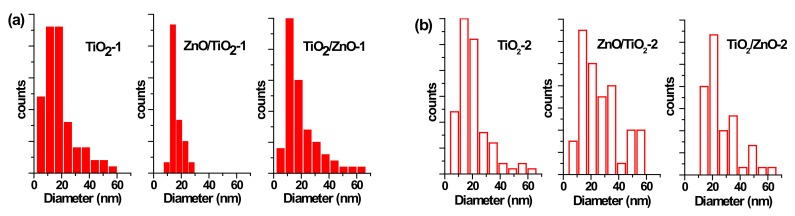
Size distribution of TiO_2_ nanoparticles (NPs) (**a**) in samples of TiO_2_-1, ZnO/TiO_2_-1, and TiO_2_/ZnO-1; and (**b**) in samples TiO_2_-2, ZnO/TiO_2_-2, and TiO_2_/ZnO-2.

**Figure 5:**
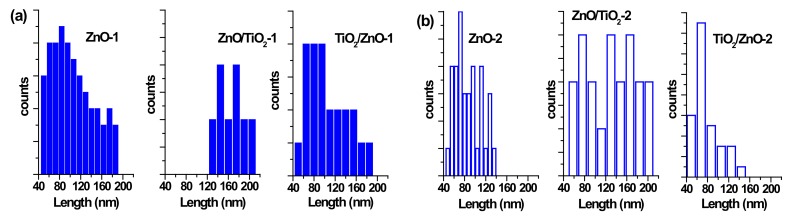
Size distribution of ZnO nanorods by length (**a**) in samples of ZnO-1, ZnO/TiO_2_-1, and TiO_2_/ZnO-1; and (**b**) in samples of ZnO-2, ZnO/TiO_2_-2, and TiO_2_/ZnO-2.

**Figure 6:**
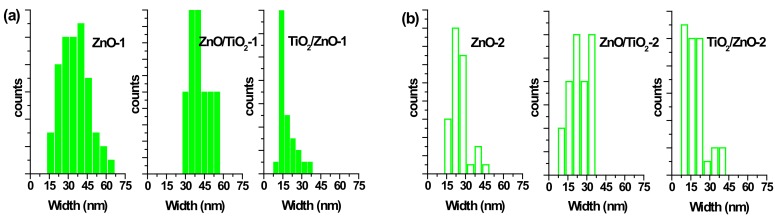
Size distribution of ZnO nanorods by width (**a**) in samples ZnO-1, ZnO/TiO_2_-1, and TiO_2_/ZnO-1; (**b**) in samples ZnO-2, ZnO/TiO_2_-2, and TiO_2_/ZnO-2.

The morphology of prepared nanostructures was examined by electron microscopy. TEM micrographs of the samples prepared at lower and higher pulse energies are presented in Figure 2 and Figure 3, respectively, as well as Figures S1 and S2 in Supplementary Materials. In general, the ZnO NPs are seen to be rod-shaped, while TiO_2_ NPs look spherical. The TEM images reveal that upon drying, ZnO nanorods are aggregated in flower-like structures irrespective of the sample, i.e. both in pure ZnO and hybrids with TiO_2_ (Figure 2a,c,d). The TiO_2_ NPs are within a wide range of sizes, depending on the sample and its preparation conditions (Figure 2b–d and Figure 3b–d). It is also seen that ZnO nanorods are well peppered with TiO_2_ NPs in both hybrids (see panels (c) and (d) in Figure 2 and Figure 3, as well as Supplementary Materials). In order to evaluate the effects of laser pulse peak power and nature of the liquid phase on the size and aspect ratio of the nanorods, TEM images were used to determine the diameter of TiO_2_ NPs, as well as the length and width of ZnO nanorods. For this purpose, several TEM micrographs were processed using the Image J software to measure particle sizes and plot the histograms presented in Figure 4, Figure 5 and Figure 6.

The particle size distribution was found to be wider when pulses with lower energy were applied to a metal plate in water, as it can be seen in Figure 5a and Figure 6a for ZnO-1. Similar material prepared at higher pulse energy (ZnO-2) shows a narrower size distribution in Figure 5b and Figure 6b. Another example, shown in Figure 4a, is the sizes of TiO_2_ NPs LAL-generated in pure water (samples TiO_2_-1 and TiO_2_/ZnO-1), which are seen to range between 5 and 65 nm. At the same time, the TiO_2_ NPs formed in ZnO suspension (ZnO/TiO_2_-1) were twice smaller (with an average diameter of 18 nm, Figure 4a) and had a narrower size distribution. This effect could result from the presence of ZnO NPs, which partially absorbed laser photons, thus partially suppressing titanium plate ablation. In parallel, ZnO nanorods obtained in the titania colloid (TiO_2_/ZnO-1) demonstrated various lengths and larger aspect ratio values (length to width), as their growth was somewhat slower under such conditions (Figure 5a and Figure 6a), while their growth rate is known to be preferential along the axial [001] direction [42]. The ZnO NPs formed in the hybrid sample of ZnO/TiO_2_-1 are seen in Figure 5a and Figure 6a to be thicker and more uniform in length in comparison with the hybrid of TiO_2_/ZnO-1, which most likely resulted from longer laser irradiation time of ZnO NPs in ZnO/TiO_2_-1. It is thus seen that at lower pulse energy, the liquid medium significantly affected the NP size and shape of both metal oxide phases (ZnO, TiO_2_), which was observed for both pure and hybrid materials.

Previously, the size and aspect ratio of ZnO nanorods prepared by millisecond-pulsed laser in water were reported to increase along with pulse width [42]. The histograms in Figure 5a are consistent with such previous results, as ZnO rods in the ZnO-1 sample showed a length up to 190 nm and a width between 15 and 65 nm. For comparison, the sample of ZnO-2 prepared with a shorter pulse duration was observed to have nanorods with shorter lengths (40–140 nm) and smaller widths (15–50 nm) (Figure 5b and Figure 6b). The ZnO nanorods that were already present in the colloid prior to the ablation of the Ti plate (in the ZnO/TiO_2_-1 sample) were heat-treated for a much longer period of time, which is believed to be the main reason for their bigger length (Figure 5a). Increased size of LAL-produced ZnO nanorods was previously reported, and the effect was explained by elevated temperatures in the reaction vessel [30,42]. It is worth noting that in the present study, the temperature increased up to 70 °C during the ablation of all samples at lower-energy pulses, while using higher-energy pulses resulted in temperatures close to 80 °C.

At the same time, changing the pulse energy seemed not to affect the produced TiO_2_ NPs significantly, as their size remained essentially unchanged in both water and ZnO suspension, in the range of 5–65 nm (Figure 4b). Most likely, the applied conditions (pulse energy) were enough to cause secondary fragmentation of titania NPs in all the prepared samples. Ablation of the Zn plate in water and in TiO_2_ suspension (ZnO-2 and TiO_2_/ZnO-2 samples) was observed to produce ZnO nanorods with similar dimensions (Figure 5b and Figure 6b); thus, the effect of pulse energy (and medium temperature) was stronger than that of the environment (pure water or colloid with titania NPs). However, prolonged irradiation of ZnO NPs during the preparation of the ZnO/TiO_2_-2 sample is seen to lead to elongated rods (up to 210 nm) with narrower width (10–35 nm) in Figure 5b and Figure 6b. Therefore, irrespective of lower or higher pulse energies used, the post-irradiation of ZnO NPs was found to generate longer ZnO nanorods (Figure 5a,b).

### 3.3. XPS Analysis

**Figure 7:**
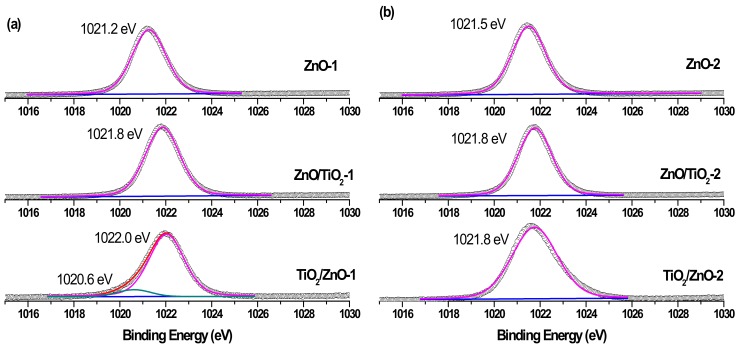
Zn 2p_3/2_ XPS spectra of samples (**a**) ZnO-1, ZnO/TiO_2_-1, and TiO_2_/ZnO-1; and (**b**) ZnO-2, ZnO/TiO_2_-2, and TiO_2_/ZnO-2. Curve-fitted experimental data are shown by the pink peak (lattice Zn^2+^) and dark cyan peak (metallic Zn).

**Figure 8:**
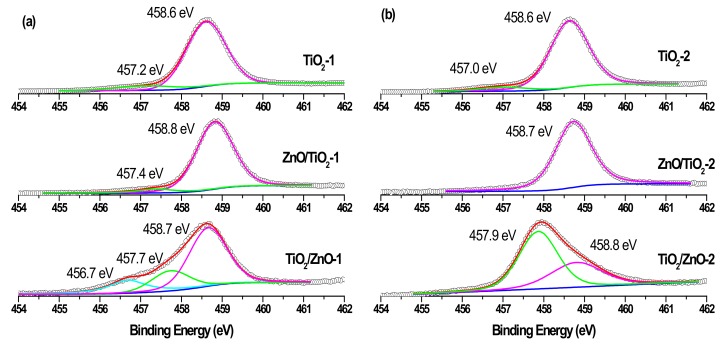
Ti 2p_3/2_ XPS spectra of samples (**a**) TiO_2_-1, ZnO/TiO_2_-1, and TiO_2_/ZnO-1; and (**b**) TiO_2_-2, ZnO/TiO_2_-2, and TiO_2_/ZnO-2. Curve-fitted experimental data are shown as pink peak (Ti^4+^ species), green peak (Ti^3+^ species), and blue peaks (Ti^2+^ species).

**Figure 9:**
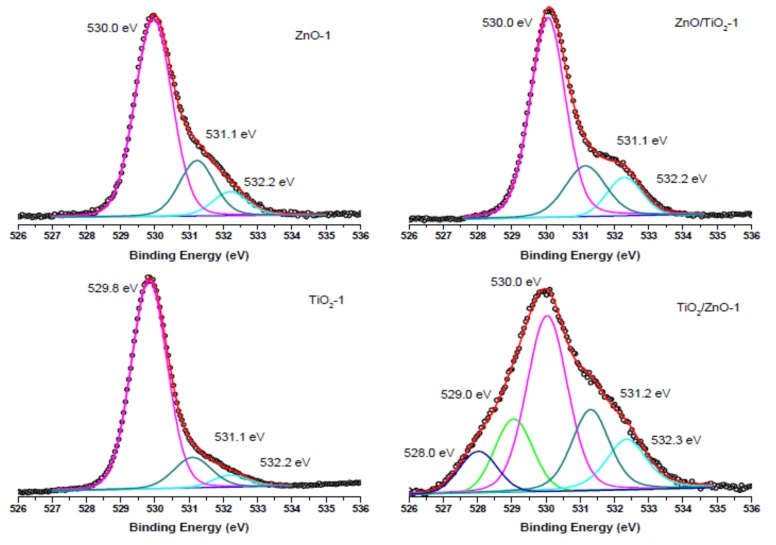
O 1s XPS spectra of samples of ZnO-1, TiO_2_-1, ZnO/TiO_2_-1, and TiO_2_/ZnO-1, all prepared with lower-energy pulses. Curve-fitted experimental data are presented as pink peak (lattice O^2−^), dark cyan peak (oxygen vacancies), blue peak (OH^-^ species), green peak (Ti^3+^-O^2−^), and dark purple peak (Ti^2+^-O^2−^).

**Figure 10:**
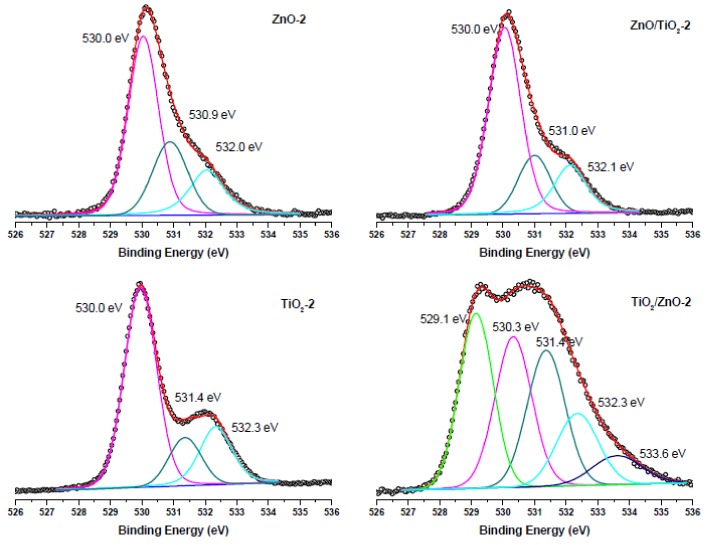
O 1s XPS spectra of samples of ZnO-2, TiO_2_-2, ZnO/TiO_2_-2, and TiO_2_/ZnO-2, all prepared with higher-energy pulses. Curve-fitted experimental data are presented as pink peak (lattice O^2−^), dark cyan peak (oxygen vacancies), blue peak (OH^−^ species), green peak (Ti^3+^-O^2−^), and navy-blue peak (chemisorbed oxygen).

The surface composition of the prepared hybrid materials was evaluated by carrying out XPS measurements and analyzing Zn 2p, Ti 2p, and O 1s peaks presented below in Figure 7, Figure 8, Figure 9 and Figure 10. The spin-orbit splitting observed between Zn 2p_3/2_ and Zn 2p_1/2_ levels in all Zn 2p XPS spectra was the same (23.0 eV), being in agreement with the literature [42,44,51]. Therefore, for convenience, only Zn 2p_3/2_ peaks are exhibited in Figure 7. A slight shift towards higher binding energies is observed for hybrid materials in comparison with pure ZnO NPs obtained at both lower and higher pulse energies (compare spectra in Figure 7a,b). This could be attributed to some electron transfer from titania to the ZnO surface when the two phases form a hybrid material.

Similarly, while the Ti 2p_3/2_ peaks in single-phase TiO_2_-1 and TiO_2_-2 are observed at 458.6 eV, those of hybrid materials are seen in Figure 8a,b to be slightly shifted towards larger binding energy and are located at 458.7–458.8 eV [20,26]. At the same time, both hybrids (TiO_2_/ZnO-1 and TiO_2_/ZnO-2) prepared under prolonged laser irradiation of TiO_2_ (which first formed during laser ablation and then was irradiated during the second stage when ZnO material was formed) demonstrate the presence of Ti^3+^ ions in their surface layer (indicated by the green peak in Figure 8a,b, bottom spectra) [18,22]. The concentration of this species is predictably higher in the material prepared at higher pulse energy (TiO_2_/ZnO-2 sample), implying that such ions form as a result of the interaction of the surface titania layer with laser photons or with laser-induced species in water. Moreover, a weak signal for the Ti^2+^ species (at 456.7 eV) was also observed in the TiO_2_/ZnO-1 sample (Figure 8a, bottom spectrum), which demonstrates the further reduction of Ti^3+^ ions [20,25].

The O 1s XPS spectra of the ZnO-1, TiO_2_-1, ZnO/TiO_2_-1, and TiO_2_/ZnO-1 samples (all prepared with lower-energy pulses) are displayed in Figure 9. The most intense peak at 530.0 eV (and 529.8 eV for TiO_2_-1) is assigned to the coordinatively saturated oxide ion in the crystal lattice of ZnO (and TiO_2_). The signals observed at higher binding energies (531.1 eV and 532.2 eV) are those from oxygen vacancies and surface hydroxide groups, respectively. At the same time, in agreement with the spectra in Figure 8a, the TiO_2_/ZnO-1 sample shows two additional peaks assigned to oxygen atoms in the Ti^3+^-O^2−^ and Ti^2+^-O^2−^ bonds.

Comparison with the corresponding O1s XPS spectra of the samples prepared with higher-energy pulses presented in Figure 10 reveals that the peaks (lattice O^2−^, oxygen vacancies and OH^−^ ions) keep their position, while the intensity resulting from oxygen vacancies in TiO_2_/ZnO-2 is higher (the peak at 531.4 eV indicated by dark cyan color), which should be explained by a greater density of oxygen defects caused by irradiation with higher energy fluence and prolonged laser-treatment of titania NPs. Furthermore, the lowest-energy peak at 529.1 eV in the spectrum of theTiO_2_/ZnO-2 sample, which is assigned to the Ti^3+^-O bond, is much stronger than that in the TiO_2_/ZnO-1 sample (compare green peaks in Figure 9 and Figure 10). This is in good agreement with the strong peak for Ti^3+^ observed for the TiO_2_/ZnO-2 sample (compare green peaks in Figure 8a,b). In addition, the O1s XPS spectrum of the TiO_2_/ZnO-2 sample (Figure 10) has a relatively high contribution of chemisorbed oxygen (navy-blue peak at 533.6 eV) [20,52].

The results of the XPS analysis imply that the surface chemistry of the samples depends on the laser parameters used, as well as on the sequence in which Ti and Zn targets were ablated. As a result, one- and two-phase (hybrid) nanomaterials were prepared with a different surface composition, including different surface defects (such as, oxygen vacancies and Ti^3+^ and Ti^2+^ species). The ability to control surface chemistry and defects, and to prepare TiO_2_-ZnO hybrid nanomaterials with different hetero-junctions opens an avenue for the preparation of hybrids with different catalytic, sensing, and/or optoelectronic properties using a simple and inexpensive technique. It is expected that such nanomaterials with a defect-rich surface will demonstrate enhanced photocatalytic and gas-sensing properties, which is a topic of further research work.

### 3.4. UV-Vis Absorption Spectra

**Figure 11:**
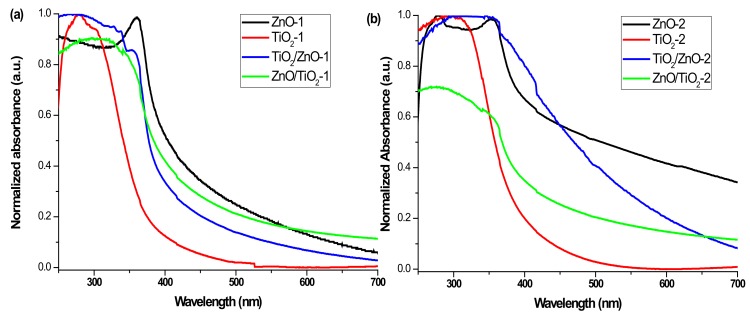
UV-vis spectra of (**a**) ZnO-1, TiO_2_-1, ZnO/TiO_2_-1, and TiO_2_/ZnO-1, and (**b**) ZnO-2, TiO_2_-2, ZnO/TiO_2_-2, and TiO_2_/ZnO-2.

Figure 11 presents the UV-vis absorption spectra of all the samples measured as freshly prepared colloids, with the nanomaterials prepared at lower and higher pulse energy being presented in panels (a) and (b), respectively. The characteristic peak of ZnO NPs is observed at 360 and 355 nm for ZnO-1 and ZnO-2, respectively (black spectra) [44]. Both show a blue shift with respect to the bulk ZnO (380 nm) [52], which resulted from a decrease in particle size. In the spectra of hybrid materials, the band for ZnO is observed as a shoulder located around 360 nm, which is weaker for the ZnO/TiO_2_-1 and ZnO/TiO_2_-2 samples, and is more pronounced for TiO_2_/ZnO-1 and TiO_2_/ZnO-2. This observation is in agreement with the above-proposed secondary irradiation to which ZnO NPs were subjected (getting modified) during ablation of the Ti target when ZnO/TiO_2_ samples were prepared. In addition, the ZnO-2 sample demonstrates a weak peak at 278 nm, which is attributed to metallic Zn, indicating some inclusions of unreacted zinc, as higher-energy pulses produced some fraction of larger molten drops of metallic zinc (Figure 11b) [42]. The absorption in the UV region (below 300 nm) observed for the hybrid samples of TiO_2_/ZnO-2 and ZnO/TiO_2_-2 (both prepared at higher pulse energy) can also be assigned to metallic Zn inclusions, in accordance with XRD patterns where weak peaks for Zn were detected (Figure 1d).

Both samples based on pure TiO_2_ NPs (TiO_2_-1 and TiO_2_-2) show pronounced absorption peaks centered at 290–300 nm (see red spectra in Figure 11a,b) [23,25,50]. At the same time, the hybrid nanomaterials reveal wide absorption bands indicating a combination of both ZnO and TiO_2_ NPs (see blue and green spectra in Figure 11a,b). The absorption maximum for the ZnO/TiO_2_-2 sample observed at 280 nm (green spectrum in panel (b)) is seen to be blue-shifted in comparison with that of the ZnO/TiO_2_-1 sample (310 nm). The peak of TiO_2_/ZnO-2 is centered at a higher wavelength (325 nm) than that of TiO_2_/ZnO-1 (290 nm) (compare blue spectra in panels (a) and (b)). Moreover, the blue spectrum in panel (b) shows a high-wavelength tale from enhanced absorption in the visible region, which can probably be explained by a defect-rich structure of TiO_2_/ZnO-2, in agreement with the XPS results presented above. Thus, the UV-vis spectra also confirm that hybrid nanomaterials of TiO_2_ and ZnO demonstrate different optical properties depending on the preparation conditions.

## 4. Conclusions

In this work, using a millisecond-pulsed laser, a series of single-phase and mixed hybrid nanomaterials based on ZnO, TiO_2_, and their mixtures were prepared. The study focused mainly on the preparation of hybrid nanomaterials of the two semiconductors, and different approaches were attempted to prepare mixed ZnO-TiO_2_ and TiO_2_-ZnO nanomaterials using laser pulses with lower and higher energy. It was shown that the sequence of metal ablation affects the composition of the hybrids, while the reaction medium and laser power influence the particle size. All the hybrid nanoparticles prepared were found to consist of ZnO nanorods and TiO_2_ nanospheres, while their sizes and surface chemistry varied depending on the processing parameters applied. The diameter of TiO_2_ nanospheres and aspect ratio of ZnO nanorods are medium-dependent when lower laser energy is used. However, higher laser energy causes secondary fragmentation of TiO_2_ nanospheres in both water and ZnO dispersion, resulting in a wider particle size distribution. Regardless of the applied laser energy, the extended irradiation time of ZnO nanoparticles (as part of hybrid material) leads to elongated nanorods. The XPS analysis clearly showed that a prolonged laser treatment of TiO_2_ nanoparticles induced their self-doping with Ti^3+^ and Ti^2+^ ions and enriched them with surface oxygen vacancies, whose density increased with increasing laser power.

## Figures and Tables

**Table 1:** Description of experimental conditions and sample notations.

Sample Notation	Laser Conditions					
	Pulse Peak Power (kW)	Pulse Width (ms)	Pulse Energy (J/pulse)	Time (min)	Liquid Medium	Plate
TiO_2_-1	1.0	2.0	2.0	30	Water	Ti
ZnO-1	1.0	2.0	2.0	30	Water	Zn
ZnO/TiO_2_-1	1.0	2.0	2.0	30 + 30	ZnO colloid *	Zn/Ti
TiO_2_/ZnO-1	1.0	2.0	2.0	30 + 30	TiO_2_ colloid *	Ti/Zn
TiO_2_-2	5.0	1.0	5.0	30	Water	Ti
ZnO-2	5.0	1.0	5.0	30	Water	Zn
ZnO/TiO_2_-2	5.0	1.0	5.0	30 + 30	ZnO colloid *	Zn/Ti
TiO_2_/ZnO-2	5.0	1.0	5.0	30 + 30	TiO_2_ colloid *	Ti/Zn

* Dispersed in water.

## Supplementary Materials

The following are available online at https://www.mdpi.com/1996-1944/13/3/719/s1, Figures S1 and S2.

## References

[1] Zhang D., Gökce B., Barcikowski S. (2017). Laser Synthesis and Processing of Colloids: Fundamentals and Applications. Chem. Rev..

[2] Amans D., Cai W., Barcikowski S. (2019). Status and demand of research to bring laser generation of nanoparticles in liquids to maturity. Appl. Surf. Sci..

[3] Zeng H.B., Du X.W., Singh S.C., Kulinich S.A., Yang S.K., He J.P., Cai W.P. (2012). Nanomaterials via laser ablation/ irradiation in liquid: A review. Adv. Funct. Mater..

[4] Niu K.Y., Yang J., Kulinich S.A., Sun J., Li H., Du X.W. (2010). Morphology control of nanostructures via surface reaction of metal nanodroplets. J. Am. Chem. Soc..

[5] Xiao J., Liu P., Wang C.X., Yang G.W. (2017). External field-assisted laser ablation in liquid: An efficient strategy for nanocrystal synthesis and nanostructure assembly. Prog. Mater. Sci..

[6] Zhang J., Claverie J., Chaker M., Ma D. (2017). Colloidal metal nanoparticles prepared by laser ablation and their applications. ChemPhysChem.

[7] Zhang J., Chaker M., Ma D. (2017). Pulsed laser ablation based synthesis of colloidal metal nanoparticles for catalytic applications. J. Colloid Interface Sci..

[8] Reichenberger S., Marzun G., Muhler M., Barcikowski S. (2019). Perspective of surfactant-free colloidal nanoparticles in heterogeneous catalysis. ChemCatChem.

[9] Zhang D., Liu J., Li P., Tian Z., Liang C. (2017). Recent advances in surfactant-free, surface-charged, and defect-rich catalysts developed by laser ablation and processing in liquids. ChemNanoMat.

[10] Feng Y., Li Z., Liu H., Dong C., Wang J., Kulinich S.A., Du X.W. (2018). Laser-prepared CuZn alloy catalyst for selective electrochemical reduction of CO_2_ to ethylene. Langmuir.

[11] Mintcheva N., Srinivasan P., Rayappan J.B.B., Kuchmizhak A.A., Gurbatov S., Kulinich S.A. (2020). Room-temperature gas sensing of laser-modified anatase TiO_2_ decorated with Au nanoparticles. Appl. Surf. Sci..

[12] Niu K.Y., Kulinich S.A., Yang J., Zhu A.L., Du X.W. (2012). Galvanic replacement reactions of active metal nanoparticles. Chem.-Eur. J..

[13] Gavrilenko E.A., Goncharova D.A., Lapin I.N., Nemoykina A.L., Svetlichnyi V.A., Aljulaih A.A., Mintcheva N., Kulinich S.A. (2019). Comparative study of physicochemical and antibacterial properties of ZnO nanoparticles prepared by laser ablation of Zn target in water and air. Materials.

[14] Mintcheva N., Aljulaih A.A., Bito S., Honda M., Kondo T., Iwamori S., Kulinich S.A. (2018). Nanomaterials produced by laser beam ablating Sn-Zn alloy in water. J. Alloys Compd..

[15] Wang H.B., Wang J.Q., Mintcheva N., Wang M., Li S., Mao J., Liu H., Dong C.K., Kulinich S.A., Du X.W. (2019). Laser synthesis of iridium nanospheres for overall water splitting. Materials.

[16] Liu P., Cai W., Fang M., Li Z., Zeng H., Hu J., Luo X., Jing W. (2009). Room temperature synthesized rutile TiO_2_ nanoparticles induced by laser ablation in liquid and their photocatalytic activity. Nanotechnology.

[17] Zimbone M., Cacciato G., Buccheri M.A., Sanz R., Piluso N., Reitano R., La Via F., Grimaldi M.G., Privitera V. (2016). Photocatalytical activity of amorphous hydrogenated TiO_2_ obtained by pulsed laser ablation in liquid. Mater. Sci. Semicond. Proc..

[18] Pan S.S., Lu W., Zhao Y.H., Tong W., Li M., Jin L.M., Choi J.Y., Qi F., Chen S.G., Fei L.F. (2013). Self-doped rutile titania with high performance for direct and ultrafast assay of H_2_O_2_. ACS Appl. Mater. Interfaces.

[19] Amin M., Tomko J., Naddeo J.J., Jimenez R., Bubb D.M., Steiner M., Fitz-Gerald J., O’Malley S.M. (2015). Laser-assisted synthesis of ultra-small anatase TiO_2_ nanoparticles. Appl. Surf. Sci..

[20] Huang C.N., Bow J.S., Zheng Y., Chen S.Y., Ho N., Shen P. (2010). Nonstoichiometric titanium oxides via pulsed laser ablation in water. Nanoscale Res. Lett..

[21] Nikolov A.S., Atanasov P.A., Milev D.R., Stoyanchov T.R., Deleva A.D., Peshev Z.Y. (2009). Synthesis and characterization of TiO_x_ nanoparticles prepared by pulsed-laser ablation of Ti target in water. Appl. Surf. Sci..

[22] García Guillén G., Shaji S., Mendivil I., Avellaneda D., Castillo G., Roy T., Garcia-Gutierrez D., Krishnan B. (2017). Effects of ablation energy and post-irradiation on the structure and properties of titanium dioxide nanomaterials. Appl. Surf. Sci..

[23] Hong S.M., Lee S., Jung H.J., Yu Y., Shin J.H., Kwon K.Y., Choi M.Y. (2013). Simple preparation of anatase TiO_2_ nanoparticles via pulsed laser ablation in liquid. Bull. Korean Chem. Soc..

[24] Singh S.C., Swarnkar R.K., Gopal R. (2009). Synthesis of titanium dioxide nanomaterial by pulsed laser ablation in water. J. Nanosci. Nanotechnol..

[25] Barreca F., Acacia N., Barletta E., Spadaro D., Curro G., Neri F. (2010). Small size TiO_2_ nanoparticles prepared by laser ablation in water. Appl. Surf. Sci..

[26] Chaturvedi A., Joshi M.P., Mondal P., Sinha A.K., Srivastava A.K. (2017). Growth of anatase and rutile phase TiO_2_ nanoparticles using pulsed laser ablation in liquid: Influence of surfactant addition and ablation time variation. Appl. Surf. Sci..

[27] Boutinguiza M., Rodríguez-González B., del Val J., Comesaña R., Lusquiños F., Pou J. (2012). Production of TiO_2_ crystalline nanoparticles by laser ablation in ethanol. Appl. Surf. Sci..

[28] Boutinguiza M., Rodríguez-González B., del Val J., Comesaña R., Lusquiños F., Pou J. (2011). Laser-assisted production of spherical TiO_2_ nanoparticles in water. Nanotechnology.

[29] Reich S., Göttlicher J., Letzel A., Gökce B., Barcikowski S., dos Santos Rolo T., Baumbach T., Plech A. (2018). X-ray spectroscopic and stroboscopic analysis of pulsed-laser ablation of Zn and its oxidation. Appl. Phys. A.

[30] Ishikawa Y., Shimizu Y., Sasaki T., Koshizaki N. (2006). Preparation of zinc oxide nanorods using pulsed laser ablation in water media at high temperature. J. Colloid Interface Sci..

[31] Kulinich S.A., Kondo T., Shimizu Y., Ito T. (2013). Pressure effect on ZnO nanoparticles prepared via laser ablation in water. J. Appl. Phys..

[32] Goto T., Honda M., Kulinich S.A., Shimizu Y., Ito T. (2015). Defects in ZnO nanoparticles laser-ablated in water-ethanol mixture at different pressures. Jpn. J. Appl. Phys..

[33] Cho J.M., Song J.K., Park S.M. (2009). Characterization of ZnO nanoparticles grown by laser ablation of a Zn target in neat water. Bull. Korean Chem. Soc..

[34] Kim K.K., Kim D., Kim S.K., Park S.M., Song J.K. (2011). Formation of ZnO nanoparticles by laser ablation in neat water. Chem. Phys. Lett..

[35] Dorranian D., Solati E., Dejam L. (2012). Photoluminescence of ZnO nanoparticles generated by laser ablation in deionized water. Appl. Phys. A.

[36] Zamiri R., Zakaria A., Ahangar H.A., Darroudi M., Zak A.K., Drummen G.P.C. (2012). Aqueous starch as a stabilizer in zinc oxide nanoparticle synthesis via laser ablation. J. Alloys Compd..

[37] Usui H., Shimizu Y., Sasaki T., Koshizaki N. (2005). Photoluminescence of ZnO nanoparticles prepared by laser ablation in different surfactant solutions. J. Phys. Chem. B.

[38] Kawabata K., Nanai Y., Kimura S., Okuno T. (2012). Fabrication of ZnO nanoparticles by laser ablation of sintered ZnO in aqueous solution. Appl. Phys. A.

[39] He C., Sasaki T., Usui H., Shimizu Y., Koshiza N. (2007). Fabrication of ZnO nanoparticles by pulsed laser ablation in aqueous media and pH-dependent particle size: An approach to study the mechanism of enhanced green photoluminescence. J. Photochem. Photobiol. A.

[40] Desarkar H.S., Kumbhakar P., Mitra A.K. (2013). One-step synthesis of Zn/ZnO hollow nanoparticles by the laser ablation in liquid technique. Laser Phys. Lett..

[41] Niu K.Y., Yang J., Kulinich S.A., Sun J., Du X.W. (2010). Hollow nanoparticles of metal oxides and sulfides: Fast preparation via laser ablation in liquid. Langmuir.

[42] Honda M., Goto T., Owashi T., Rozhin A.G., Yamaguchi S., Ito T., Kulinich S.A. (2016). ZnO nanorods prepared via ablation of Zn with millisecond laser in liquid media. Phys. Chem. Chem. Phys..

[43] Kondo T., Sato Y., Kinoshita M., Shankar P., Mintcheva N., Honda M., Iwamori S., Kulinich S.A. (2017). Room temperature ethanol sensor based on ZnO prepared via laser ablation in water. Jpn. J. Appl. Phys..

[44] Mintcheva N., Aljulaih A.A., Wunderlich W., Kulinich S.A., Iwamori S. (2018). Laser-ablated ZnO nanoparticles and their photocatalytic activity towards organic pollutants. Materials.

[45] Abbas K.N., Bidin N. (2017). Morphological driven photocatalytic activity of ZnO nanostructures. Appl. Surf. Sci..

[46] Kubiak A., Siwinska-Ciesielczyk K., Jesionowski T. (2018). Titania-based hybrid materials with ZnO, ZrO_2_ and MoS_2_: A review. Materials.

[47] Lee B.-H., Nakayama T., Tokoi Y., Suzuki T., Niihara K. (2011). Synthesis of CeO_2_/TiO_2_ nanoparticles by laser ablation of Ti target in cerium (III) nitrate hexahydrate (Ce(NO_3_)_3_·6H_2_O) aqueous solution. J. Alloys Compd..

[48] Gondal M.A., Ilyas A.M., Fasasi T.A., Dastageer M.A., Seddigi Z.S., Qahtan T.F., Faiz M., Khattak G.D. (2015). Synthesis of green TiO_2_/ZnO/CdS hybrid nano-catalyst for efficient light harvesting using an elegant pulsed laser ablation in liquids method. Appl. Surf. Sci..

[49] Zhang X., Yuan J., Zhu J., Fan L., Chen H., He H., Wang Q. (2019). Visible light photocatalytic performance of laser-modified TiO_2_/SnO_2_ powders decorated with SiC nanocrystals. Ceram. Int..

[50] Gondal M.A., Ilyas A.M., Baig U. (2016). Pulsed laser ablation in liquid synthesis of ZnO/TiO_2_ nanocomposite catalyst with enhanced photovoltaic and photocatalytic performance. Ceram. Int..

[51] Moulder J.F., Stickle W.F., Sobol P.E., Bomben K.D. (1992). Handbook of X-ray Photoelectron Spectroscopy.

[52] Zhang X., Qin J., Xue Y., Yu P., Zhang B., Wang L., Liu R. (2014). Effect of aspect ratio and surface defects on the photocatalytic activity of ZnO nanorods. Sci. Rep..

