# Supplementary material for: Hybrid TiO2-ZnO Nanomaterials Prepared Using Laser Ablation in Liquid
Source: Materials (Basel). 2020 Feb 5;13(3):719. doi: 10.3390/ma13030719 (PMC7040934; doi:10.3390/ma13030719)
Supplement: Supplementary file 1 [file materials-13-00719-s001.pdf]

materials

MDPI

Article

Hybrid TiO_2-ZnO Nanomaterials Prepared Using Laser Ablation in Liquid

Neli Mintcheva [1,2,*], Shigeru Yamaguchi [3] and Sergei A. Kulinich [1,4,5,*]

[1] Research Institute of Science and Technology, Tokai University, Hiratsuka, Kanagawa 259-1292, Japan
[2] Department of Chemistry, University of Mining and Geology, Sofia 1700, Bulgaria
[3] Department of Physics, Tokai University, Hiratsuka, Kanagawa 259-1292, Japan; shigeru@keyaki.cc.u-tokai.ac.jp
[4] Department of Mechanical Engineering, Tokai University, Hiratsuka, Kanagawa 259-1292, Japan
[5] School of Natural Sciences, Far Eastern Federal University, Vladivostok 690041, Russia
* Correspondence: nnmintcheva@mgu.bg (N.M.); skulinich@tokai-u.jp (S.K.)

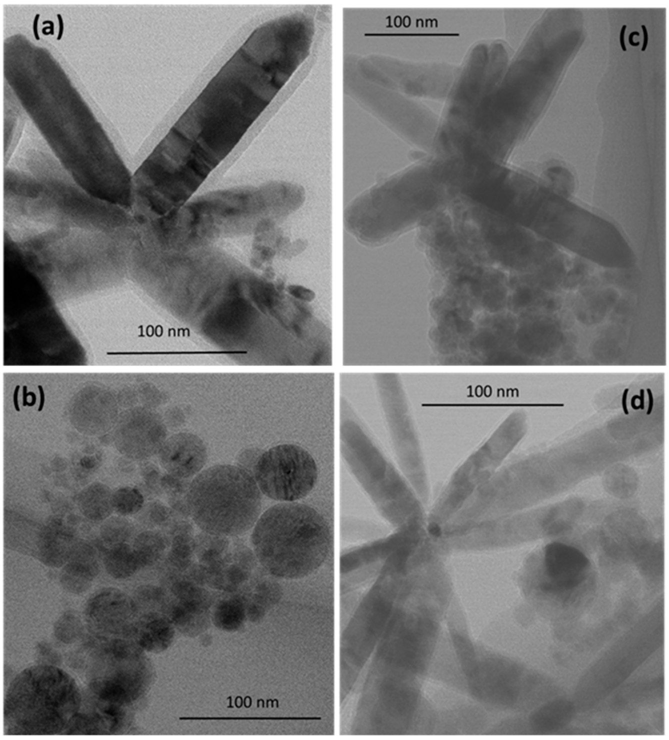

Figure 1. TEM images of samples prepared at lower pulse energy: (**a**) ZnO-1, (**b**) TiO_2-1, (**c**) ZnO/TiO2-1 and (**d**) TiO_2/ZnO-1.

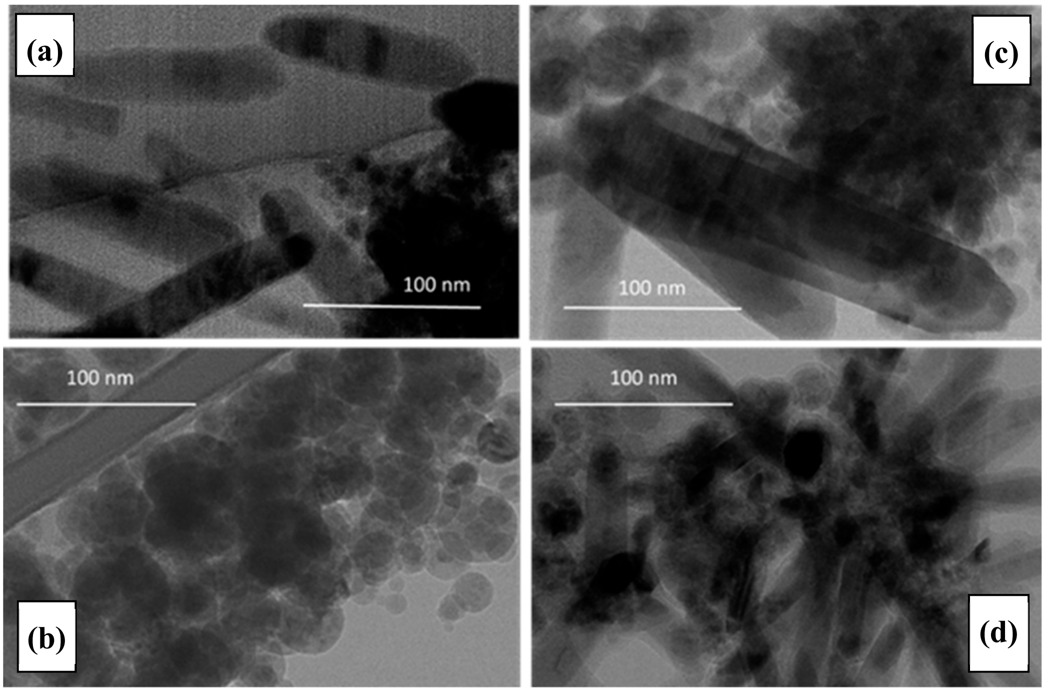

Figure 2. TEM images of samples prepared at higher pulse energy: (**a**) ZnO-2, (**b**) TiO_2-2, (**c**) ZnO/TiO_2-2 and (**d**) TiO_2/ZnO-2.

© 2020 by the authors. Licensee MDPI, Basel, Switzerland. This article is an open access article distributed under the terms and conditions of the Creative Commons Attribution (CC BY) license (http://creativecommons.org/licenses/by/4.0/).
